# Mode hybridisation strategies for subwavelength sound attenuation in miniaturised Helmholtz resonators

**DOI:** 10.1038/s41598-026-46693-6

**Published:** 2026-04-01

**Authors:** R. Domingo-Roca, A. Feeney, J. C. Jackson-Camargo, J. F. C. Windmill

**Affiliations:** 1 University of Strathclyde, Glasgow, Scotland, UK; 2 University of Glasgow, Glasgow, Scotland, UK

**Keywords:** Engineering, Materials science, Optics and photonics, Physics

## Abstract

Controlling wave propagation is a topic of fundamental importance across many areas of physics. Photonic crystals have proven highly effective for this purpose. However, because they rely on Bragg interference, they typically require relatively large structural dimensions. Metamaterials, on the other hand, attenuate sound by breaking the mass-density law, and can exhibit deep subwavelength attenuation. Miniature acoustic resonators capable of broadband, tuneable sound manipulation are essential for next-generation compact noise-control systems. Here, we investigate metamaterials for subwavelength operation based on resonant unit cells composed by Helmholtz resonators. We interrogate the introduction of structural compliance as means to decrease the operating frequency of the device. Starting from a conventional HR, this work shows that geometric scaling alone is constrained by enhanced thermoviscous losses, limiting achievable miniaturisation. By incorporating compliant elements in the unit cell, it is possible to couple acoustic and mechanical degrees of freedom, producing hybridised modes that enable frequency reduction and direction-dependent while remaining within the deep subwavelength regime. Complex frequency analysis reveals that compliance and viscoelastic losses jointly govern the motion of resonant poles and zeroes, offering a unified framework to interpret the balance between miniaturisation, absorption, and bandwidth. The proposed metamaterials achieve subwavelength at a factor of *λ*/38, and their design is made possible by innovative multi-material light-based 3D-printing techniques. The results establish compliance-driven modal hybridisation as a general principle for engineering compact and tuneable acoustic systems, bridging traditional HRs and emerging soft metamaterials. This framework offers a physically grounded route toward scalable, broadband, and directionally responsive acoustic systems.

## Introduction

Attenuating sound in small-scale devices is a non-trivial endeavour. Acoustic waves easily penetrate thin walls due to a combination of low mass and low structural resistance. According to the mass-density law, the transmission of sound occurs proportionally to the inverse of density and thickness of the material, and the frequency of the incoming sound wave^1,2^, therefore demanding dense, thick materials for efficient sound attenuation. Similarly, phononic crystals (engineered, periodic composite structures analogous to atomic lattices designed to manipulate and control acoustic waves) result in large devices that seriously restrain the range of applications in the low-frequency regimes, where wavelengths are large.

Metamaterials, on the contrary, display spatial scales typically much smaller than the wavelength^3,4^. Acoustic metamaterials derive their advanced functionalities through designed subwavelength structures (unit cells patterned in space) that govern their macroscopic properties^5,6^. Metamaterials have been widely investigated for their potential to display effective unusual properties such as high refractive indices^7,8^ and negative effective properties^9–12^, which are usually studied under the approach of the effective medium theory and experimentally tested from the far field^13^. In airborne acoustics, the collective excitation of the unit cells leads to acoustic surface waves that propagate through periodic arrays of air-filled cavities^14–18^, membranes^19–21^, or Helmholtz resonators^19,22^, and are localised at the surface they propagate along. When the effective properties are negative, stop bands are formed where sound is deeply attenuated at frequencies proximal to the unit cell’s resonance, enabling metamaterials to break the mass-density law. However, the frequency and bandwidth of simple air-filled resonators are typically limited by the dimensions of the unit cell, and decreasing the operational frequency is linked to a volume increase.

This motivates the present work; we aim to design a unit cell with an acoustic response that remains low-frequency, subwavelength and broadband without compromising on its volume. This aligns with one of the main aims of current acoustic engineering: tackling the miniaturisation challenge. Helmholtz resonators (HRs), which manipulate wave propagation through local resonances, remain excellent candidates for this purpose given their subwavelength nature that enables them to manipulate sound efficiently and compactly. HRs have become foundational elements in modern acoustic engineering, finding applications in architectural acoustics^23,24^, noise mitigation^25,26^, and aeroacoustics^27^. However, classical HRs are inherently narrowband and lack the tunability required for modern applications demanding multi-frequency, broadband, or directional response.

Helmholtz resonators consist of a cavity (of volume *V*_*c*_) connected to ambient space through a narrow neck (of cross-sectional area $\:{A}_{N}$), and behave as lumped acoustic systems where the open end of the neck radiates sound, providing radiation resistance and a radiation mass. Their resonant frequency, $\:{f}_{HR}$, is well understood after the works of Helmholtz, Rayleigh, Sondhauss and Wertheim^28^ (Eq. 1).

1 $$\:{f}_{HR}=\frac{c}{2\pi\:}\sqrt{\frac{{A}_{N}}{{V}_{C}{L}_{eff}}}$$

Where *c* is the wave speed in the propagating medium and $\:{L}_{eff}={H}_{N}+\alpha\:$, with $\:{H}_{N}$ being the neck’s height, and *α* the end correction factor (which considers that the effective length of the vibrating air column in the neck is slightly longer than its geometric length, a consequence of the kinetic energy of the air flow at the openings). The magnitude of *α* depends on the geometry around the opening, and requires numerical calculation for complex geometries (although two idealised cases provide 1.7*R*_*N*_ and 1.4*R*_*N*_, for flanged and unflanged neck terminations, respectively^29,30^, with *R*_*N*_ being the radius of the neck).

Over the years, researchers have modified HRs to achieve low-frequency operation; coupling multiple necks and cavities^31–33^, introducing coiled necks^34,35^, combination with microperforated plates^36^, and integrating microlattice linings^37,38^ are a few examples. These systems display multiple resonant modes and complex modal interactions, broadening the effective bandwidth of the unit cell and enabling angular selectivity or directional filtering, although their fundamental response still relies on neck/cavity geometries and still demanding large volumes, particularly for low-frequency operation.

Integration of membranes within HRs, whether as backplates, boundary elements, or cavity splitters, has been an area of interest since it introduces a distinct class of acoustic dynamics^18,39–41^. The membranes contribute additional elastic compliance and facilitate structural-acoustic coupling, resulting in hybridised resonant modes that are unattainable in conventional rigid-walled designs, as shown in the works from Horowitz^42^ and Choi and Jeon^39^. These structures offer compelling advantages, including anisotropic attenuation and directional sensitivity without the need for external actuation or control. However, these works highlight inaccuracies between theoretical and experimental results due to the application of in-plane tension either as an inadvertent effect of clamping, or as an intrinsic difficulty of fine-tuning membrane tension. Moore et al. addressed this issue by means of 3D-printing plastic rings on pre-laid polyethylene sheets, providing a periodic, uniform medium of membrane-backed cavities, and demonstrating sound control at operable frequencies below those dictated by the physical depth of the sample at λ/10 subwavelength. Despite the promising results, this technique might be limited when using other 3D-printing approaches (i.e., light-based) without temperature control or higher resolution. Casarini et al. investigated how to integrate membranes into HRs via stereolithography of polyethylene glycol diacrylate, achieving subwavelength attenuation of λ/12 in unit cells as small as 4.2 mm in diameter. More interestingly, Casarini et al. demonstrated coupling of different *M*$\:\times\:$*N* arrays, and investigated the directional response of the system, moving away from conventional laboratory testing and envisioning real-World applications of these metamaterials. Sabat et al. and Magariyachi et al. also investigated directionality of HRs via coupled systems^43,44^, suggesting that frequency degeneracy, coupling strength, and directional response depend on the relative position of the HR openings in the far field.

This work presents an experimental study that aims to tackle the miniaturisation challenge through the introduction of multi-material compliant unit cells based on HRs. The unit cells are realised via multi-material 3D-printing and without relying on post-assembly or post-processing protocols. The introduction of compliant elements on the neck and multi-port systems proposed in this work differ fundamentally from traditional multi-neck architectures: while necks behave, primarily, as inertial channels for oscillating air masses, other types of acoustic ports act as coupled oscillators that not only react to incoming sound but also radiate secondary waveforms that interfere constructively or destructively with the primary acoustic field. This introduces new opportunities for tackling the miniaturisation challenge, tailoring the directional response, enhancing tunability, and achieving multi-modal energy trapping in compact, passive structures. The introduction of compliant elements within the unit cell offers a mechanism to lower the operating frequency without compromising on its physical size. These systems could prove transformative in areas such as stealth, acoustics, noise control in complex environments, and bio-inspired directional hearing technologies.

## The conventional Helmholtz resonator

**Fig. 1:**
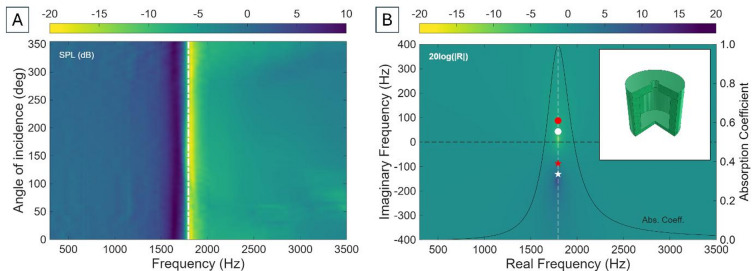
(**A**) Experimental sound pressure level (SPL) of a conventional Helmholtz resonator (HR) as a function of frequency and incidence angle. The white dot-dash line shows the Helmholtz resonance obtained using Eq. (1). (**B**) Representation of the 20log(|R|) in the complex frequency plane for the HR. The red dot and star represent the zero and the pole, respectively, obtained through the lossless ($\:X=0$) low frequency approximation [Eqs. (3) and (4)]. The white dot (zero) and star (pole) introduce losses into the system ($\:X>0$). The numerical results (viridis colourbar) introduce realistic radiation and thermoviscous losses (*R*_*r*_, *R*_*w*_), rather than introducing a $\:X$ value from a toy model. Absorption coefficient is theoretically determined. The inset shows the CAD file of the unit cell.

The acoustic response of conventional HRs has been widely studied^45–47^. In brief, the acoustic behaviour of a HR is often modelled as an acoustic circuit comprising an inertial neck and a compliant cavity connected in series, with impedances ${Z}_{N}=i\omega\:{L}_{N}$ and ${Z}_{C}=-i/\omega\:{C}_{V}$, respectively (with neck inertance ${L}_{N}=\rho\:{L}_{eff}/{A}_{N}$ and cavity compliance ${C}_{V}={V}_{C}/\rho\:{c}^{2}$)^48^. The moving fluid in the neck radiates sound into the surrounding medium in the same manner as an open-ended pipe, offering both radiation ($\:{R}_{r}$) and thermoviscous ($\:{R}_{w}$) resistance as ${R}_{r}={\rho}_{0}c{k}^{2}{A}_{N}/2\pi$ and ${R}_{w}=2mc{\alpha}_{w}$, where *m* is the mass of air displaced by the neck, and ${\alpha}_{w}$ is the combined absorption coefficient for wall losses, given by^29^:

2 $${\alpha}_{w}=\frac{1}{{R}_{N}c}{\left(\frac{\eta\:\omega\:}{2\rho}\right)}^{1/2}\left(1+\frac{\gamma\:-1}{\sqrt{Pr}}\right)$$

where $\:\omega\:$ is the angular frequency, $\:\rho\:$ is the density of air, $\:\eta\:$ is the kinematic viscosity of air, $\:\gamma\:$ is the specific heat ratio of air, and $\:Pr$ is the Prandtl number. The impedance of the neck when taking these into account becomes $\:{Z}_{N}=i\omega\:{L}_{N}+{R}_{N}/{S}_{N}^{2}$. Under plane wave incidence, the pressure is given by $\:{p}_{1}={p}_{0}\mathrm{e}\mathrm{x}\mathrm{p}\left[i\omega\:(t+\:{\delta\:}_{1}/\mathrm{c})\mathrm{c}\mathrm{o}\mathrm{s}\left(\theta\:\right)\right]$^48^. where $\:{\delta\:}_{1}$ is the geometrical centre of the HR (${\delta}_{1}=({H}_{C}+{L}_{eff})/2$), and $\:\theta\:$ is the angle of incidence of the wave. This formulation assumes that the resonator responds uniformly to incoming waves from all directions, which holds true for idealised resonators in symmetric configurations. Translating impedance boundary conditions to the time domain requires expressing the acoustic impedance in a form compatible with causal, transient dynamics. This is achieved by taking the Laplace transform of the impedance function, which necessitates extending its definition from real angular frequencies to the full complex frequency plane ($s=\sigma\:+i\omega\:=2\pi\:({F}_{r}+i{F}_{i})$). In this framework, the poles and zeroes of the impedance encode the resonant behaviour of the system (including bandwidth, damping, and energy storage) while ensuring physical causality and stability. This formulation provides a deeper understanding of resonance characteristics and enables extension to more complex resonant systems. The acoustic response of a 3$\:\times\:$3 array of cylindrical HRs with cylindrical necks of dimensions *R*_*N*_ = 1 mm, *H*_*N*_ = 2 mm, cavity radius *R*_*C*_ = 5 mm, and cavity height *H*_*C*_ = 10 mm, is shown in Fig. 1.

Figure 1A shows the existence of the stop band at 1833 Hz, matching the theoretical Helmholtz resonance predicted by Eq. (1) (white dot-dash line). Given that the lateral distance between the centres of the units is 14 mm, the metamaterial is subwavelength λ/13.4. 20 dB attenuation dB is observed at the resonance frequency, with a pronounced dip resulting from impedance matching between the resonator and the surrounding medium. This impedance matching maximises energy transfer into the cavity, where it is subsequently dissipated through thermoviscous losses. After resonance, the HR impedance becomes stiffness-dominated, breaking the impedance matching and preventing efficient energy transfer, resulting in significantly less energy being absorbed. Figure 1A shows that sweeping over angle of incidence displayed an omnidirectional response at Helmholtz resonance, in line with the subwavelength symmetry of the resonator^22,49^. This behaviour is a direct fingerprint of the pole-zero structure of the HR in the complex frequency plane. If one understands the total impedance of the HR as the impedance of the neck and cavity in series ($\:{Z}_{HR}={Z}_{N}+{Z}_{C}$), it is straightforward to obtain, theoretically, the reflection, $\:R=({Z}_{HR}-{Z}_{0})/({Z}_{HR}+{Z}_{0})$, and absorption, $\:\alpha\:=1-{\left|R\right|}^{2}$ coefficients as a function of $\:s=2\pi\:({F}_{r}+i{F}_{i})$. The numerical solution of this formulation is displayed in Fig. 1B. The red dot and star in Fig. 1B represent the analytical positions of the zero and pole, respectively, using the lossless ($\:X=0$) low-frequency approximation toy model proposed by Romero-García et al.^46^ (Eqs. (3) and (4)).

3 $$\:{k}_{zero}=\sqrt{{k}_{HR}^{2}-\frac{{(1-X)}^{2}}{4{\beta\:}^{2}}}+i\frac{1}{2\beta\:}(1-X)$$

4 $$\:{k}_{pole}=\sqrt{{k}_{HR}^{2}-\frac{{(1-X)}^{2}}{4{\beta\:}^{2}}}-i\frac{1}{2\beta\:}(1-X)$$

where $\:{k}_{HR}$ is the resonance wavenumber of the HR, *β* is a parameter related with leakage, and $\:X$ is a constant real part representing the introduction of losses in the system. In the lossless case, the system is purely reactive – the poles and zeroes lie exactly on the imaginary axis, meaning there is no leakage and resonance is undamped, leading to perfect absorption. In the lossy case, the poles and zeroes appear as complex conjugate pairs in the frequency plane, consistent with causality and reciprocity (fundamental properties arising from the intrinsic nature of the wave equation). The real part of the complex frequency corresponds to the resonance frequency, while the imaginary part corresponds to the decay or leakage rate of acoustic energy to the environment^46^. The presence of losses in the system ($\:X>0$) introduces a real part in the impedance of the system (notice that the impedance of an ideal HR is purely imaginary, since $\:{Z}_{HR}={Z}_{N}+{Z}_{C}=i\omega\:{L}_{N}-i/\left(\omega\:{C}_{C}\right)$), causing the dipole to shift downward in the $\:Im\left(f\right)$ axis (Fig. 1B). However, it must be noted that the sign of the imaginary parts of poles and zeroes depends on the time convention^46^. When these losses balance the leakage of the system, the imaginary part becomes zero and the real part coincides with the resonance frequency of the HR, producing perfect absorption. In single-port systems, the condition of zero reflection indicates critical coupling, which can occur at real frequencies when the intrinsic (dissipative) losses exactly balance the radiation leakage to the external medium^50^. Similarly, when gain is introduced such that it exactly compensates radiation losses, a pole moves to the real-frequency axis, marking the lasing threshold^50^. In practice, HRs are inherently non-Hermitian since their function relies on dissipation to achieve enhanced absorption. In Fig. 1B, the pole corresponding to the Helmholtz resonance lies close to the real axis, indicating low intrinsic losses and a high-Q resonance. In the idealised symmetric formulation, losses are neglected, and oscillations are sustained indefinitely, resulting in perfect symmetric pole-zero pairs. When losses are introduced, the pole shifts down, meaning that the system’s natural oscillation decays exponentially with time (i.e., resonance is stable but damped). The peak in the absorption spectrum aligns with the real part of this pole, corroborating that the resonant mode is the primary contributor to energy dissipation. Figure 1B shows excellent agreement between analytical and numerical calculations of poles and zeroes, also aligning with experimental results. Note: from now onwards, only the lossy dipole will be displayed to aid visualisation of losses.

Theoretical calculations yield $\alpha\:=0.9925$, leading to theoretical SPL values at resonance of − 21.25 dB. Figure 1A provides experimental dB values at resonance of − 19.04 dB, matching well with theoretical calculations. The 2.2 dB difference might arise from microphone sensitivity/calibration, mismatches on plane-wave assumption, or resonator leakage.

### Effects and limitations of size and geometry

**Fig. 2:**
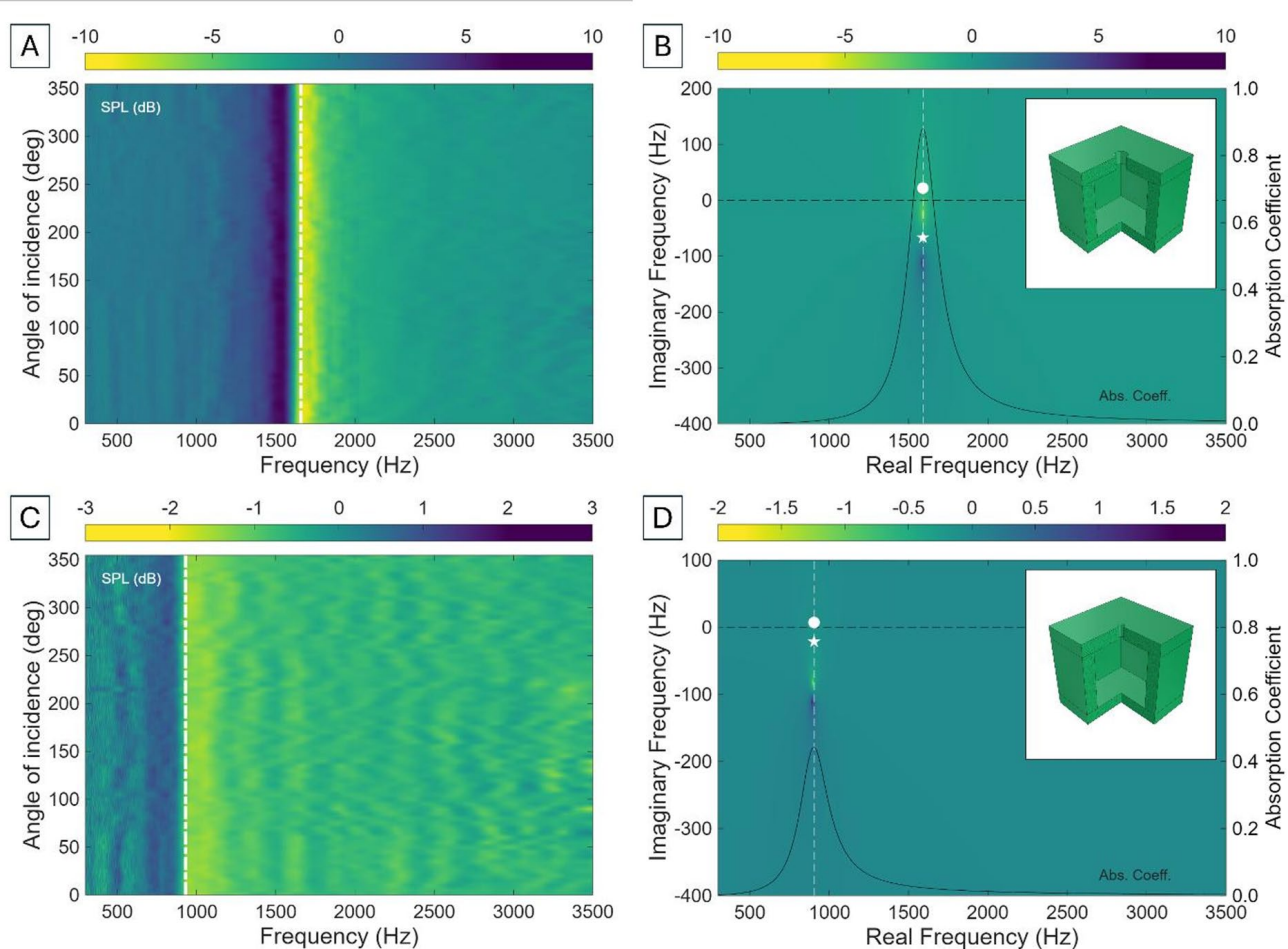
(**A**) Experimental sound pressure level (SPL) of a conventional Helmholtz resonator (HR) with rectangular cavity cross-section as a function of frequency and incidence angle. The white dot-dash line shows the theoretical Helmholtz resonance (Eq. 1). (**B**) Corresponding representation of the 20log(|R|) in the complex frequency plane. The white dot and star represent, respectively, the lossy zero and the pole, and the numerical results (viridis colourbar) introduce realistic radiation and thermoviscous losses. (**C**) and (**D**) show the same representations for ta HR of rectangular cross-section, with a neck of radius 0.5 mm. The insets show the CAD file of the unit cell.

It is immediately clear from Eq. (1) that reducing the resonance of a HR requires either increasing the effective cavity volume or lengthening the neck, two modifications that are incompatible with the principle of miniaturisation that guides this work. However, there remain two alternative strategies to address the miniaturisation challenge without altering the overall dimensions of the unit cell: (i) increasing the enclosed volume while maintaining comparable outer dimensions (e.g., replacing a cylindrical cavity with a parallelepiped cavity of square cross-section $\:{2R}_{N}$), and (ii) reducing the radius of the neck. Figure 2 shows the experimental and theoretical results of implementing these modifications, where the cylindrical cavity has been replaced by a parallelepiped cavity of square cross-section area of side $\:{2R}_{C}\stackrel{\scriptscriptstyle\mathrm{def}}{=}W=10\: \mathrm{mm}$.

These modifications preserve the overall subwavelength scale of the device while increasing the effective cavity volume relative to the cylindrical case. The Helmholtz resonance (Eq. 1) shifts downward compared to the cylindrical case, demonstrating the effectiveness of this approach while preserving a single-neck architecture and avoiding additional post-processing. Changing the geometry of the cavity (Fig. 2A, B) leads to a stop band at 1660 Hz (λ/15 subwavelength), and a decrease of the theoretical $\:\alpha\:$ value to 0.8796, a drop with respect to the cylindrical case attributed to increased thermoviscous boundary losses near the cavity walls. Moreover, the square geometry introduces corner effects, which can trap vorticity and thermal gradients that lead to localised dissipation compared to smooth cylindrical geometries. This behaviour is reflected in the pole-zero distribution, where the zero begins to cross into the region $\:Im\left(f\right)<0$, indicating that the anti-resonance (cancellation point) also decays over time.

Similarly, Fig. 2C and D present the experimental and theoretical implications of reducing the *R*_*N*_ from 1 mm to 0.5 mm while keeping the cavity as in Fig. 2A and B. The experimentally measured stop band appears at 984 Hz and matches well with the theoretical resonance predicted by Eq. (1), leading to λ/25 subwavelength and demonstrating strong miniaturisation potential. However, this approach introduces imporant trade-offs: a narrower neck increases thermoviscous losses, thereby reducing the quality factor of the resonance and broadening the bandwidth. This is advantageous for applications demanding broadband attenuation, although absorption remains considerably low (~ 2 dB). This behaviour is reflected in the pole-zero structure of the acoustic system, with both the pole and zero lying deep within the $\:Im\left(f\right)<0$ region and indicating that energy loss dominates the system and causes both the resonance and anti-resonance to decay rapidly rather than persist. Consequently, the system exhibits smoother but shallower absorption instead of a sharp bandgap.

The acoustic resistance in the neck of a HR arises primarily from viscous and thermal boundary layers forming along the neck walls. As *R*_*N*_ decreases, the surface-to-volume ratio increases, causing a larger fraction of the oscillating air to interact with the boundaries, thereby enhancing dissipation. Simultaneously, the smaller neck cross-section increases the effective acoustic mass, shifting the resonance frequency downward (see Eq. (1)), and further supporting miniaturisation. The combined effect of these mechanisms is a reduction in quality factor, manifested as a broader resonance with diminished peak attenuation. This decrease in maximum absorption reflects the energy dissipated at the neck through viscous and thermal processes – energy that would otherwise be more efficiently coupled into the cavity, producing deeper but narrower attenuation. The theoretical absorption coefficient is now considerably lower than in the previous cases ($\alpha\:=0.4423$), in good agreement with experimental measurements.

Both approaches highlight the importance of geometrical tailoring as an effective pathway to achieve compact, low-frequency operation in HRs without violating the constraints of miniaturisation. Reducing *R*_*N*_ can be beneficial in a two-fold manner, enabling both strong sub-wavelength performance and broader band attenuation. However, it is clear that continuously decreasing *R*_*N*_ eventually leads to dominant acoustic losses, whereby the system becomes overdamped and overall attenuation efficiency vanishes. In addition, achieving micron-scale neck diameters introduces significant manufacturing and post-processing challenges, limiting the practical extent of this miniaturisation strategy.

This baseline study serves as a reference point for subsequent modifications, in which compliant elements and multi-port configurations are introduced with the aim to further lower the operational frequency while maintaining compactness. The three systems presented thus far exhibit an omnidirectional acoustic response at the bandgap frequency, a consequence of their single-port nature where a single aperture simultaneously serves as the input and output for sound. In contrast, and as we shall see in the following sections, multi-port resonators might exhibit directional responses since their boundary conditions and radiation impedances depend on incidence angle (a coupling effect absent in the single-port, low-wavelength limit^48^.

## Membrane-coupled Helmholtz resonators

Modifications of cavity geometry and neck dimensions offer straightforward strategies to tune HRs. However, these approaches are fundamentally limited by the trade-off between resonance frequency reduction and increased thermoviscous losses. A more transformative route towards miniaturisation and enhanced functionality lies in the introduction of compliant boundary elements that hybridise the conventional HR dynamics with structural degrees of freedom.

**Fig. 3:**
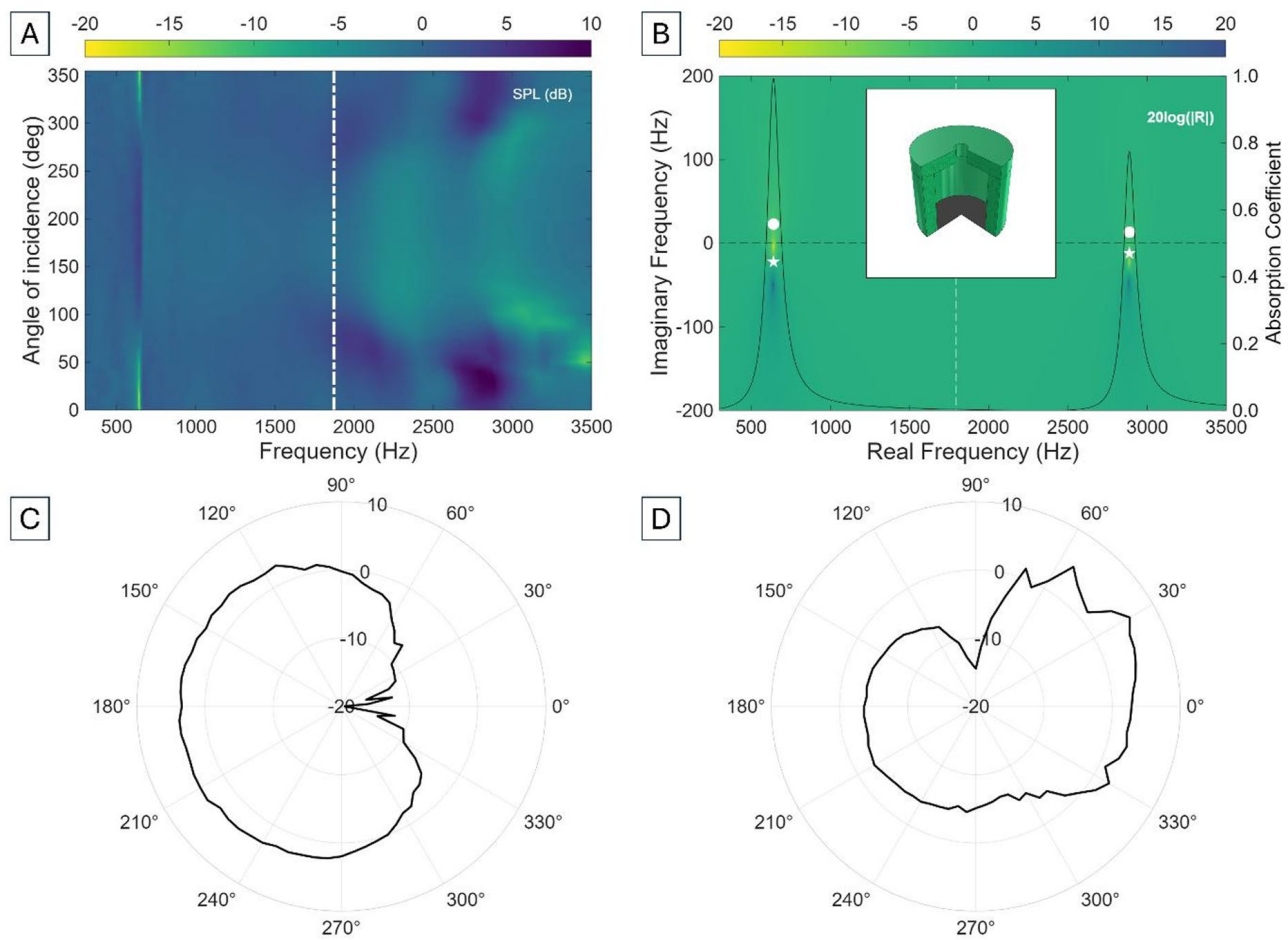
(**A**) Experimental sound pressure level (SPL) of a membrane-coupled Helmholtz resonator (M-HR) with cylindrical cavity cross-section as a function of frequency and incidence angle. The white dot-dash line shows the unmodified Helmholtz resonance (Eq. 1). (**B**) Corresponding representation of the 20log(|R|) in the complex frequency plane. The white dot and star represent, respectively, the lossy zero and the pole, and the numerical results (viridis colourbar) introduce realistic radiation and thermoviscous losses. (**C**) and (**D**) show the directional response of the system at *f* = 643 Hz (λ/38), and *f* = 3017 Hz, respectively (λ/8). The inset shows the CAD file of the unit cell.

Membrane-coupled HRs (M-HRs) incorporate a thin elastic membrane that seals the cavity opening, replacing the rigid termination in classical designs. The membrane introduces an additional elastic compliance and effective mass, which couple with the acoustic compliance of the cavity and inertance of the neck^48^. This hybridisation produces two distinct resonant modes; a M-HR constitutes a system of two dynamically coupled acoustic oscillators (the HR cavity-neck mode and the membrane vibrational mode). Their mutual coupling via the cavity pressure field leads to hybridised acoustic eigenmodes when the coupling strength exceeds the intrinsic damping. The hybrid mode frequencies and modal character are governed by both amplitude and phase relationships between the cavity volume oscillation and membrane displacement, with in-phase and out-of-phase motion corresponding to the lower and upper hybrid branches, respectively. The resulting modal splitting lowers the effective resonance frequency well below that of the conventional HR because of the mass load of the air in the cavity-neck^48^, while also providing new opportunities for anisotropic response. The compliant membrane acts as a tuneable boundary condition; its restoring force modifies the cavity pressure dynamics while its inertia introduces an additional oscillatory pathway for energy exchange between the acoustic and structural subsystems. The presence of the membrane alters dissipation pathways: structural damping within the membrane and coupling between membrane vibrations and cavity losses contribute to enhanced energy absorption. Depending on membrane tension (3–20 N/m), the hybrid system can be tailored to perform at different subwavelength regimes^51^. Figure 3 shows the experimental and theoretical results of introducing a membrane into the unit cell.

Figure 3 shows the hybridisation of the stop band as a consequence of the system responding to acoustic pressures both at the neck and membrane. This stop band hybridisation leads to two distinct solutions, one above and one below the conventional HR resonance. Experimental bandgaps (for tension 3.5 N/m) are found at 643 Hz and 3017 Hz, providing excellent agreement with theoretical stop bands at 642 Hz and 2890 Hz, and enabling subwavelength performance at *λ*/38 and *λ*/8, respectively although their appearance relies on incidence angle (Fig. 3A). This configuration can be studied from a neck and cavity connected in series, and a membrane and a cavity connected in parallel, resulting to a total system impedance of $\:{Z}_{M-HR}={Z}_{CM}+{Z}_{N}$, where $\:{Z}_{CM}={Z}_{C}{Z}_{M}/({Z}_{C}+{Z}_{M})$. Since the membrane has both mass and stiffness, and it resists motion under pressure, its total acoustic impedance is given by $\:{Z}_{M}=i\omega\:{L}_{M}+1/\left(i\omega\:{C}_{M}\right)+{R}_{M}$. It must be noticed that both membrane compliance and resistance ($\:{C}_{M}$ and $\:{R}_{M}$) depend on membrane’s tension, such that increasing the applied stress stiffens the membrane and shifts the stop bands up in frequency. The asymmetric configuration of the M-HR makes the two acoustic ports couple through the cavity and the phase difference between the pressures acting on the neck and the membrane determines whether their contributions interfere constructively or destructively in the radiated field^48,52,53^, giving rise to a cardioid directional pattern depending on frequency and port separation (Fig. 3C and D).

Theoretical calculations yield absorption peaks of 0.9920 and 0.7721, agreeing well with experimentally-measured SPL values of − 19.62 dB and − 5.69 dB at 643 Hz and 3017 Hz, respectively. The dipole representation in the complex frequency plane (Fig. 3B) shows that the first stop band achieves almost perfect attenuation due to its proximity to the real frequency axis, in line with other works that have investigated membrane-based systems for near-perfect absorption^54–56^. The second dipole is placed further away from the real frequency axis, with the zero crossing over the $\:Im\left(f\right)<0$ region and indicating that the anti-resonance decays over time, similarly to Fig. 2D and leading to inefficient sound absorption. This representation highlights how modal hybridisation shapes the coupling between the membrane, cavity, neck, and environment, providing deeper insight into the physical mechanisms underpinning absorption strength.

## Introducing additional compliance

**Fig. 4:**
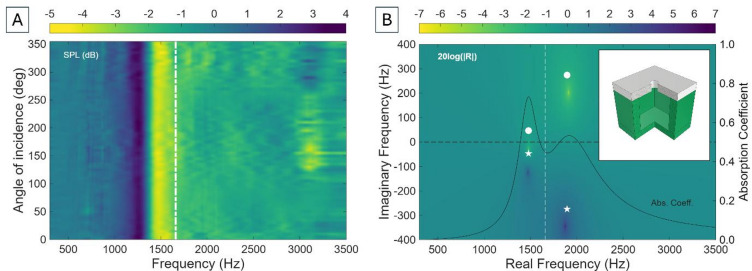
(**A**) Experimental sound pressure level (SPL) of a compliant neck Helmholtz resonator (CN-HR) with square cross section as a function of frequency and incidence angle. The white dot-dash line shows the corresponding unmodified Helmholtz resonance (Eq. 1). (**B**) Corresponding representation of the 20log(|R|) in the complex frequency plane. The white dot and star represent, respectively, the lossy zero and the pole, and the numerical results (viridis colourbar) introduce realistic radiation and thermoviscous losses (*R*_*r*_, *R*_*w*_). The inset shows the CAD file of the unit cell.

One of the key features of M-HRs is the introduction of an additional compliant term. However, $\:{C}_{M}$ directly depends on membrane tension, which can be difficult to control and replicate, particularly when dealing with small systems. There are, however, other ways to introduce additional compliance into the acoustic system^57^. One such approach is to fabricate the wall where the neck is located using an elastomeric material, such that the neck is not purely reflective but, instead, its compliance ($\:{C}_{E}$) contributes to the resonance of the system. This system will be referred to as compliant neck HR, CN-HR. This configuration can be easily achieved via multi-material 3D-printing, which facilitates fabrication of miniature complex systems with high precision without relying on manual adjustments or post-processing steps (see Methods). Figure 4 shows the experimental and theoretical results of introducing additional compliance to the neck of the HR.

This is the first case in which differences are observed between the experimental and theoretical results. While two theoretical stop bands are expected at 1465 Hz (*λ*/17) and 1945 Hz (*λ*/13), only a one clear stop band can be experimentally measured at 1405 Hz, after which SPL remains at a constant − 2 dB. Following the rationale from the previous section, one could be tempted to think that sound attenuation should be directional due to the presence of a second acoustic port. However, while the neck wall can deform, it does not constitute an independent radiating port. Unlike in the M-HR case, there is no spatial offset separation between the ports, such that phase-driven interference patterns cannot be created. Hence, although the neck compliance alters the local acoustic impedance, it does not introduce a second directional radiation channel.

Figure 4B reveals two distinct resonant modes arising from the hybridisation between conventional cavity-neck dynamics and the additional compliance introduced by the elastomeric neck wall. Since the second peak is predicted to lead to absorption values of 0.5329, that would mean only a few dB attenuation, which could explain the presence of only one peak (experimentally). The comparison between theoretical and experimental results also highlights a systematic discrepancy at higher frequencies, which is attributed to additional structural damping mechanisms in the elastomeric material that are not fully captured in the theoretical model. Elastomers dissipate energy as heat when strained, contributing additional loss compared to stiff plastics. Elastomeric materials are well known to exhibit viscoelastic behaviour, such that their elastic modulus becomes complex and frequency-dependent (${E}^{*}\left(\omega\:\right)={E}^{{\prime}}\left(\omega\:\right)+iE^{\prime \prime}\left(\omega\right)$)^58,59^. The loss factor ${E}^{{\prime \prime}}/E^{\prime}$ controls dissipation and is, typically, much larger in elastomers (10^− 1^−1) than in hard plastics (10^− 3^−10^− 2^)^60–62^. Moreover, for thick or constrained regions, there is viscous flow and relaxation in shear and bulk modes, not just elastic bending. Lastly, elastomers typically radiate sound power less efficiently than stiff plastics, meaning that structure-to-air transmission can be lower even if internal damping is high. This can be accounted for by introducing the complex elastic modulus, $\:{E}^{*}\left(\omega\:\right)$ into the theoretical model. However, at this point only conventional confined compression $\:E$ values of this elastomer are available (2.58 ± 0.12 MPa, see Methods), which, for the time-being, provide good enough predictions of the stop band frequencies.

**Fig. 5:**
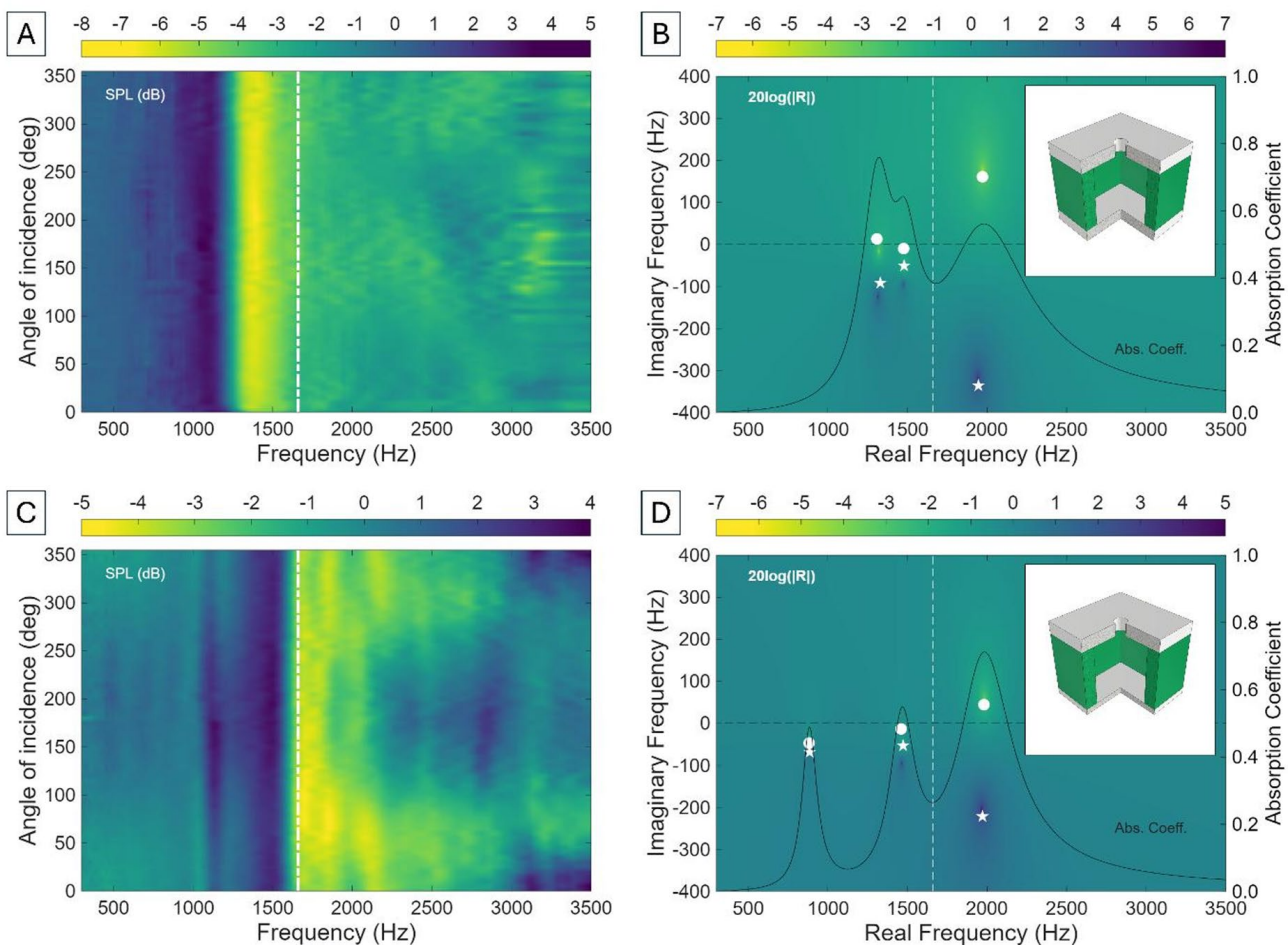
(**A**) Experimental sound pressure level (SPL) of a compliant neck Helmholtz resonator with rectangular cavity cross-section and a 2 mm elastomeric backplate (P-CN-HR) as a function of frequency and incidence angle. The white dot-dash line shows the theoretical Helmholtz resonance (Eq. 1). (**B**) Corresponding representation of the 20log(|R|) in the complex frequency plane. The white dot and star represent, respectively, the lossy zero and the pole, and the numerical results (viridis colourbar) introduce realistic radiation and thermoviscous losses. (**C**) and (**D**) show the same representations for a P-CN-HR with a 1 mm backplate.

While not being the most efficient approach to tackle miniaturisation (provides only around 200 Hz decrease in stop band frequency with respect to the corresponding unmodified HR), this acoustic system opens the doors to introducing additional compliances into the unit cell to further decrease the operating frequency by means of multi-material 3D-printing and without the need for manual assembly or post-processing. By following this route, it seems natural to decrease the *compliance of the neck*. Doing so implies decreasing the neck’s mass, which in this particular case is linked to a decrease of its length, which would increase the operating frequency rather than decreasing it. The next strategy investigates the introduction of additional compliance by introducing an elastomeric backplate in the unit cell while maintaining a *compliant neck* (Fig. 5).

Figure 5A, B shows the experimental and theoretical results of a CN-HR with a 2 mm elastomeric backplate (P-CN-HR). In this case, three peaks can be theoretically found (1340 Hz, 1475 Hz, and 1965 Hz) as a consequence of the presence of three acoustically reactive elements. However, Fig. 5A demonstrates that only one stop band is experimentally measured at 1316 Hz, reaching − 8 dB, matching the theoretical predictions. These results can be explained due to the proximity of the two first peaks, and the low attenuation achieved by the third peak (attributed to the losses within the elastomer, which are not introduced in the theoretical model).

Addition of a 2 mm elastomeric backplate has successfully demonstrated that introducing additional compliance in this manner leads to a reduced stop band frequency without compromising its size. Hence, one can keep reducing the thickness of the backplate to increase compliance (since $\:{C}_{P}\propto\:1/h$, where $\:h$ is plate thickness) and further reduce the operating frequency. Figure 5C, D shows the experimental and theoretical results of decreasing the thickness of the backplate to 1 mm. These results show that it is possible to reduce the operating stop band frequency down to 1014 Hz (*λ*/24), although it only attenuates to − 2 dB, which appears to agree with the theoretical calculations. A second, bigger dip can be measured at 1614 Hz, and a third one can be observed at 2080 Hz (displaying directionality), matching the theoretical model.

In the M-HR, the flexible membrane has, by definition, no flexural rigidity and responds strongly to small pressure differences, resulting in substantial out-of-plane motion and strong coupling with the neck aperture, yielding a cardioid-like radiation pattern. In contrast, when the membrane (60 μm) is replaced by a thick plate (2 mm), the restoring force becomes bending-dominated, reducing displacement amplitude by several orders of magnitude and suppressing pressure differentials across the cavity. Consequently, both the compliant neck and the plate move nearly in phase with the internal cavity pressure, leading to an omnidirectional response. This effect becomes weaker as the plate’s thickness decreases.

Taken together, these results highlight a crucial insight: while elastomeric compliance offers a scalable route to tune Helmholtz-type systems without the drawbacks of membrane pre-tensioning, the high internal damping intrinsic to elastomers introduces significant trade-offs between miniaturisation and absorption depth. Nonetheless, compliant-element hybridisation can be achieved in an assembly-free manner. This opens the door to systematically introducing multiple compliant terms into a single device, exploiting modal hybridisation and internal material dissipation to balance compactness, broadband response, and tuneability.

## Conclusions

This work explores a series of strategies to tackle the acoustic miniaturisation challenge using Helmholtz resonators. Beginning with the conventional HR, the study established a theoretical and experimental framework grounded in lumped-element and complex frequency plane analyses. The conventional HR exhibited the expected single, omnidirectional resonance, corresponding to the well-known Helmholtz frequency, and a near-perfect absorption coefficient ($\:\alpha\:=0.99$) under critical coupling conditions.

Geometrical modifications were then investigated as a route for resonance tuning. Increasing cavity volume led to moderate resonance reduction and a noticeable decrease in absorption (due to thermoviscous boundary losses and corner-induced dissipation). Conversely, reducing neck radius proved more effective in lowering the resonant frequency, though at the cost of quality-factor and low absorption. These confirmed that geometric modifications inherently trade resonance depth for miniaturisation, as viscous and thermal losses become dominant at smaller neck dimensions.

A more transformative approach was then demonstrated through the integration of compliant elements into the resonator system. The membrane-coupled HR (M-HR) introduced an additional elastic compliance and inertance into the system, hybridising the classical cavity-neck resonance with structural dynamics. This coupling produced two distinct resonant modes corresponding to different interactions between the components of the system. This modal hybridisation introduced anisotropic and angle-dependent responses, enabling directional selectivity. However, the dependence on membrane pre-tension introduced variability and limited reproducibility, representing a key obstacle to scalable fabrication.

To overcome these practical challenges, elastomer-based compliant HRs were developed using multi-material 3D-printing. By replacing the rigid neck wall with an elastomeric interface (compliant neck HR, CN-HR), additional compliance was introduced in a controlled, assembly-free manner. The resulting system exhibited modest reductions in resonance frequency, but proved effective in demonstrating scalable introduction of compliance into HRs without relying on fine-tuned membrane tension.

Further refinement was achieved by adding an elastomeric backplate into the unit cell. This system introduced a third reactive element, producing three hybridised resonant modes (only apparent at low enough backplate thicknesses). The lowest mode, corresponding to the thinner plate, achieved significant frequency reduction without increasing overall size, confirming that sequential addition of compliant elements enables progressive miniaturisation through modal hybridisation. However, high intrinsic damping in elastomeric materials significantly reduced absorption. These observations underline a fundamental trade-off between compliance-driven miniaturisation and dissipative losses intrinsic to soft materials.

In summary, this study demonstrates that introducing structural compliance enables systematic reduction of Helmholtz resonance frequencies and enhanced design flexibility without sacrificing compactness. While geometric scaling alone offers limited benefits due to enhanced boundary losses, hybrid acoustic-structural systems unlock new degrees of freedom for tuning, directionality, and broadband response. While the trade-off between miniaturisation and absorption depth remains governed by the balance between reactive coupling and dissipative mechanisms, this strategy has demonstrated the possibility to achieve subwavelengths of λ/25.

## Materials and methods

### Fabrication of acoustic metamaterials

The single-material Helmholtz resonators were fabricated using a PRUSA SL1S 3D printer (Prague, Czech Republic) equipped with a 405 nm LED light source. The samples were designed via computer aided design (CAD) in Autodesk Inventor and exported as STL files, which were posteriorly imported into PRUSASlicer. The STL files were then sliced at 100 μm layer thickness (*h*) without any supports nor pads and using the predefined settings (2.5 s per layer, initial exposure time of 25 s) for the commercially available PRUSA Tough Transparent Green (TG) resin (*E* = 2 GPa). After 3D-printing, the samples were thoroughly washed in isopropyl alcohol under sonication until all excess resin was removed. Removal of resin trapped within the HRs was aided by using a compressed air gun. Finally, the samples were UV post-processed for 10 min in the PRUSA Curing and Washing Machine to ensure crosslinking of any residual uncured functional groups.

**Fig. 6:**
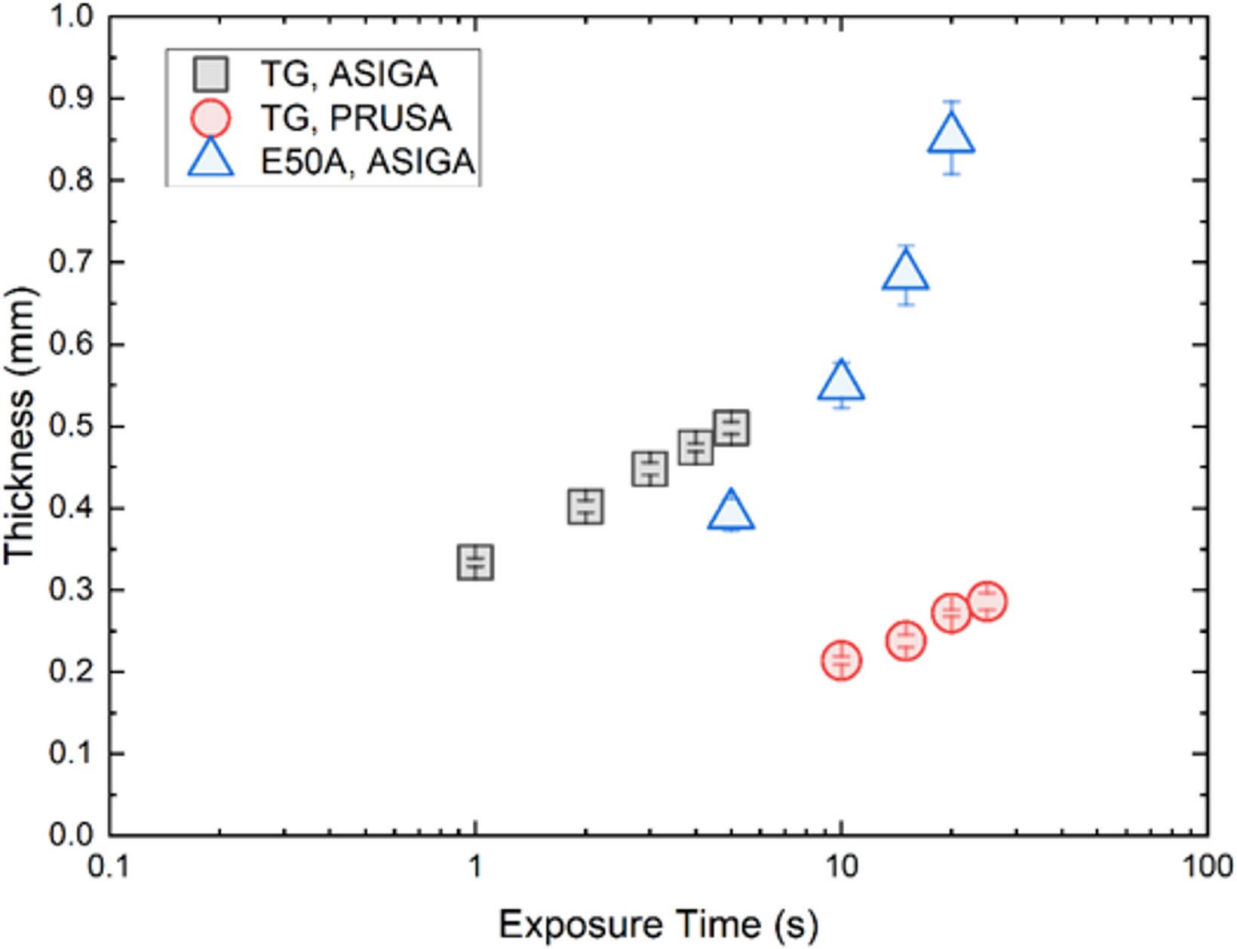
3D-printing calibration plots for the commercially-available resins PRUSA Transparent Green (TG), and FormLabs Elastomer 50A (E50A) in both 3D printers used in this work (PRUSA, ASIGA). Symbols represent average measurements (N = 5), and bars standard deviation. Fitting thickness as a function of exposure time using the Beer’s law provides the 3D-printing parameters for each resin^63^.

Multi-material metamaterials were fabricated using an ASIGA MAX27 3D printer using the same commercially available material (following material calibration, see Fig. 6). Sample slicing was done in the ASIGA Composer software, and different 3D-printing regions were defined (PRUSA TG: *I* = 31 mW/cm^2^, *h* = 0.1 mm, *t* = 0.2 s. Elastomer 50 A (FormLabs): *I* = 31 mW/cm^2^, *h* = 0.1 mm, *t* = 2 s). Once the first region had finished, the printing process was stopped, the platform zeroed vertically, and the 3D-printed part carefully washed with isopropyl alcohol while still attached to the build platform. The resin tank was then swapped with another containing a different resin (alternating between Elastomer 50 A and PRUSA TG), and the 3D-printing resumed until completion. This process was repeated as regions/materials required. Upon completion, the multi-material part was removed from the build block, cleaned with isopropyl alcohol, and cured in the UV chamber (analogously to the single material case). The dimensions of each unit cell are displayed in Table 1.

**Table 1:** Dimensions of each unit cell, including Helmholtz resonator (HR), membrane-coupled HR (M-HR), compliant neck HR (CN-HR), and backplate + CN-HR (P-CH-HR). All unspecified walls were 2 mm thick.

	HR	HR Sq. 1	HR Sq. 2	M-HR	CN-HR	*P*-CN-HR 1	*P*-CN-HR 2
Neck Radius, *R*_*N*_ (mm)	1	1	0.5	1	1	1	1
Neck Height, *H*_*N*_ (mm)	2	2	2	2	2	2	2
Cavity Type	Cyl.	Square	Square	Cyl.	Square	Square	Square
Cavity Radius, *R*_*C*_ (mm)	5	-	-	5	-	-	-
Cavity Side (mm)	-	10	10	-	10	10	10
Cavity Height, *H*_*C*_ (mm)	10	10	10	10	10	10	10
Membrane Thickness (µm)	-	-	-	60	-	-	-
Plate Thickness (mm)	-	-	-	-	-	2	1

### Acoustic testing

**Fig. 7:**
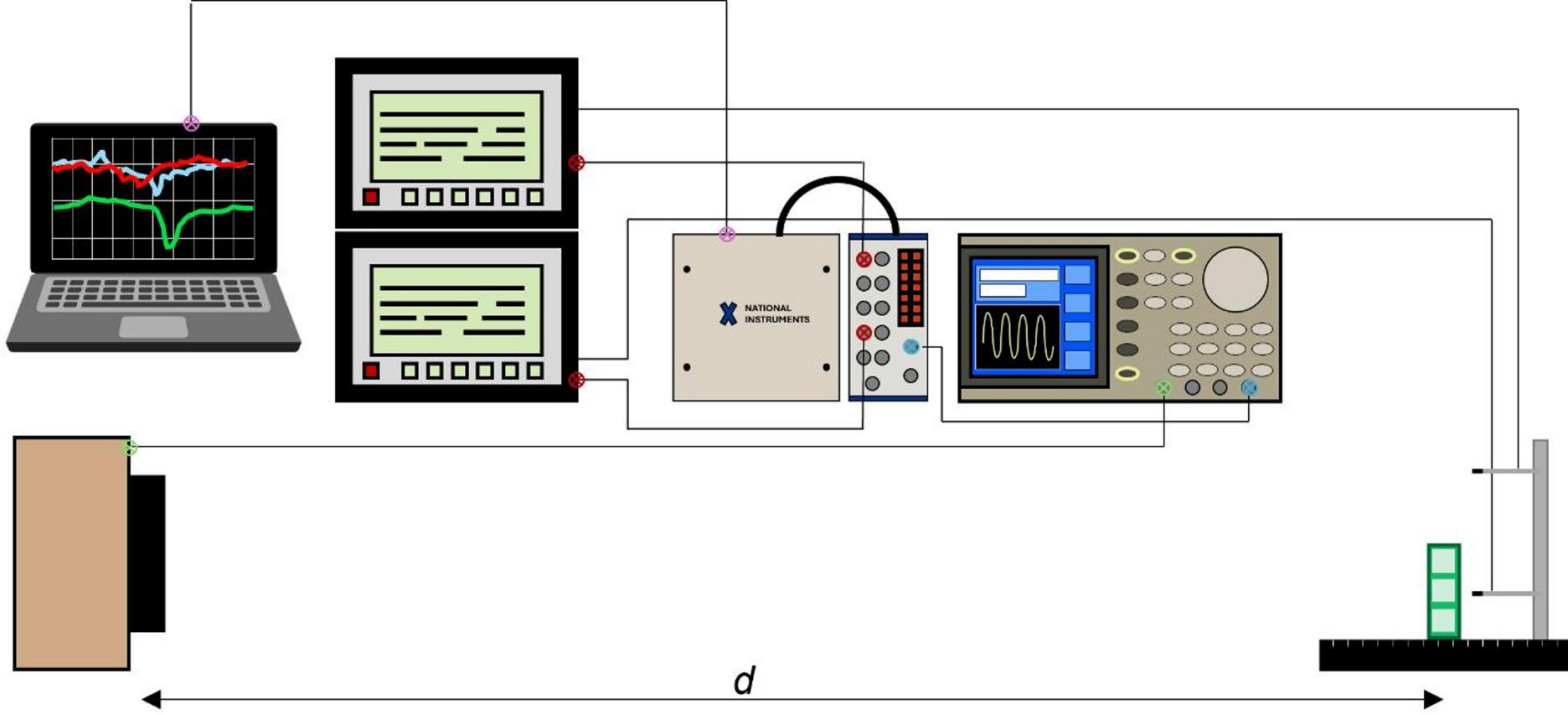
Schematic of the used experimental set up. A loudspeaker (placed a distance d from the sample, in the far field) is connected to a function generator (green circle-cross) that plays an 800 ms periodic sweep from 300 Hz to 3500 Hz. Two microphones record the acoustic response by the sample, each connected to its own pre-amplifier, and connected to the data acquisition dashboard (red circle-cross). Data acquisition is triggered to the signal generator (blue circle-cross), and the data is visualised in a laptop via a user-made MATLAB tool (pink circle-cross) that subtracts the sound pressure level (SPL) registered by each microphone.

The acoustic response of the acoustic metamaterials was recorded using two calibrated Bruel & Kjaer (4138-A-015, B&K, Naerum, Denmark) 1/8” pressure-field microphones, each connected to a pre-amplifier (WH-3219), calibrated using the B&K Type 4231 sound calibrator, conforming to the IEC/EN 60,942 (2017) Class 1, and ANSI S1.40-2006. The microphones transmitted the acquired signal to a laptop running a MATLAB session-based interface via a National Instruments data acquisition system. The microphone-pre-amplifier system was externally TTL triggered with a positive slope to the signal generator. A periodic sweep (800 ms, 10 V_pp_) between 300 Hz and 3500 Hz was generated with a function generator (Tektronix AFG3102) and emitted by a loudspeaker (DaytonAudio, ND105-4 4”) placed in the far-field (1.7 m) from the samples. The samples were mounted on a rotating stage and measured by the neck and outside its nearfield^19,22^. A reference signal was acquired using a solid block of the same dimensions than the measured MM, and both signals (in dB) were subtracted to obtain the normalised sound transmission. This reference signal is acquired to isolate the resonant behaviour of the samples from geometric scattering and mounting artifcats. Measurements were taken from 0^o^ to 360^o^ in steps of 5^o^. A schematic of the set up is shown in Fig. 7.

### Acoustic coupling in 1 × 3 arrays

**Fig. 8:**
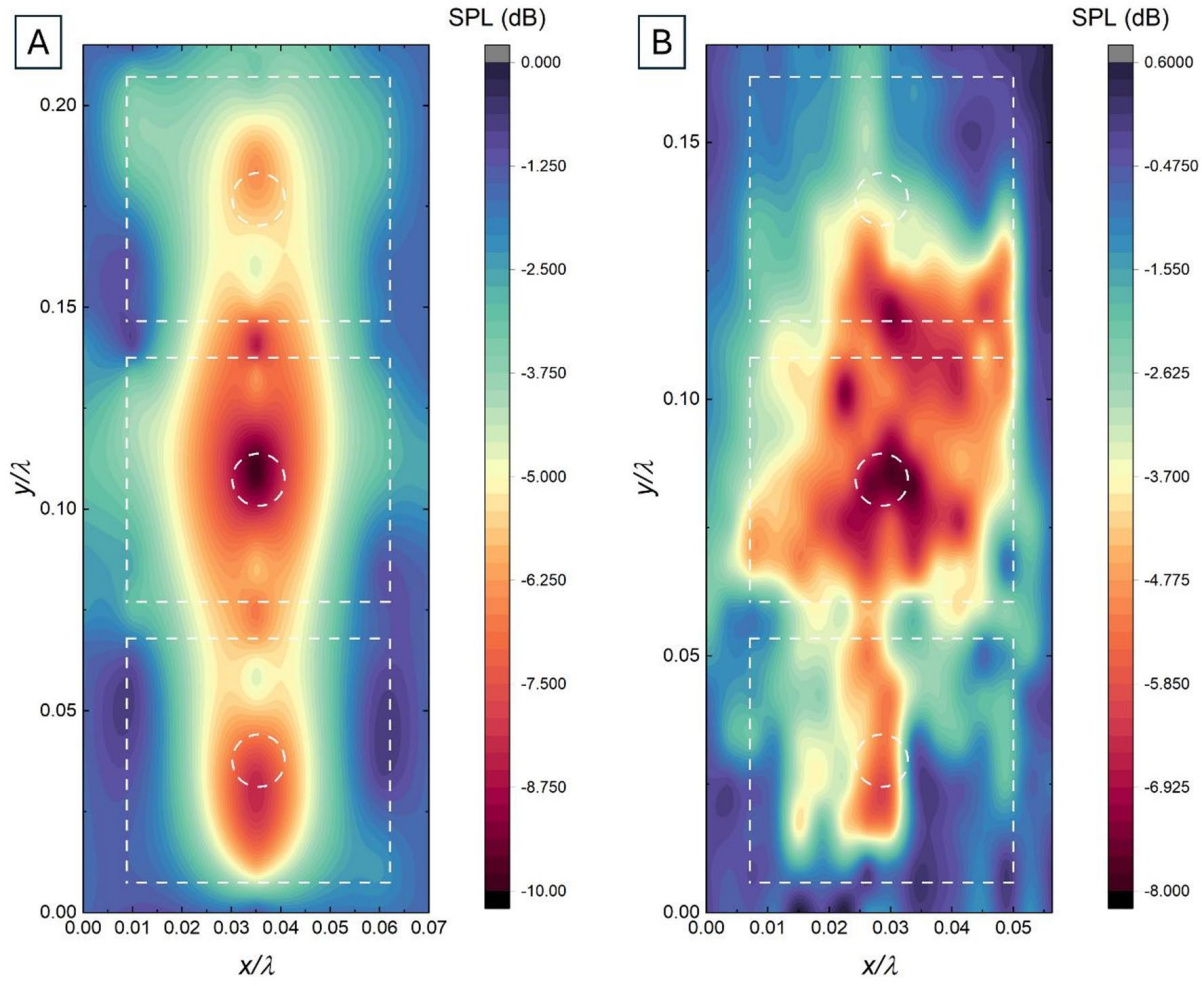
Sound pressure level (SPL) measured for (**A**) square Helmholtz resonator at 1660 Hz, and (**B**) 2 mm elastomeric backplate compliant neck Helmholtz resonator (P-CN-HR) at 1316 Hz. The white dashed lines show the physical geometry of the resonators.

Given the build size of the ASIGA MAX27 3D printer (52 × 29 × 75 mm), our resonators were limited to 1 × 3 arrays, since multi-material 3D-printing cannot be achieved otherwise. As such, it is important to determine if their acoustic response couples between neighbouring cells. This was measured by using the experimental set up previously described, and mounting the microphones on a linear stage, such that their position can be controlled with high precision and ensuring that their relative position remains the same. Acoustic measurements were taken in 2 mm steps (leading to a total of 150 points), and the results are shown in Fig. 7. (Fig. 8A corresponds to HR Sq. 1, and Fig. 8B corresponds to the P-CN-HR 1).

The SPL distribution for the HR Sq. 1 array (Fig. 8A) exhibits three well-defined minima located at the neck openings, with a largely symmetric pressure field about the vertical centreline and a smooth spatial decay away from each cavity. The response remains strongly localised within each resonator, and the spatial continuity of the background field indicates weak but finite inter-resonator interaction mediated through the surrounding acoustic domain. Overall, the array behaves predominantly as a set of discrete Helmholtz-type elements, with coupling that appears limited to nearest neighbours and primarily evanescent in character.

The P-CN-HR configuration (Fig. 8B) exhibits a more heterogeneous and asymmetric pressure distribution, with SPL features extending laterally and across unit cell boundaries. Although individual SPL minima remain identifiable, the pressure field is less spatially confined, suggesting reduced mode localisation. The presence of a compliant neck and elastomeric backplate introduces structural compliance and degrees of freedom, enabling hybrid acoustic-structural resonances that couple more efficiently. This appears to increase energy exchange between neighbouring resonators and the emergence of collective mode behaviour, rather than purely localised Helmholtz-type responses. The more complex SPL patterns and reduced spatial localisation suggest that the resonators are no longer acting as isolated resonators, but instead participate in coupled lattice dynamics characteristic of metamaterial behaviour.

Field asymmetries between the top and bottom resonators are observed in both samples. These may partly arise from a slight misalignment of the acoustic source relative to the sample and may be further influenced by viscoelastic damping in the elastomeric components, which can introduce spatially varying losses.

### Mechanical testing of Elastomer 50 A

3D-printed Elastomer 50 A disks (10 mm in diameter, 4 mm in thickness, *N* = 6) were subjected to unconfined, uniaxial compression tests using the MACH-1 (Biomomentum Inc., Canada) mechanical testing system equipped with a 250 N load cell. The samples were tested at room temperature using a flat, stainless steel indenter (12.5 mm diameter) in a cylindrical chamber (37 mm in diameter). The thickness (*h*) of each sample was measured using a pre-defined pipeline in MACH-1. Prior to compression, the elastomer was pre-loaded to an amplitude of 1% *h*, at 0.4% s^− 1^ deformation rate to ensure contact between plate and sample. Each sample was then compressed to 15% strain at 5% increments relative to *h*. After each 5% compression ramp, a 1 min hold phase was implemented. All compression steps were performed at a deformation rate of 0.1% s^− 1^ (relative to *h*). The elastic modulus (*E*) was calculated from the slope of the linear region of the stress-strain curve for each ramp. The results are displayed in Table 2.

**Table 2:** Diameter, thickness, and elastic modulus (*E*) of 6 3D-printed disk-shaped samples of Elastomer 50 A. The average of *E* is reported, together with standard deviation.

Sample	Diameter (mm)	Thickness (mm)	E (MPa)
1	10.02	4.36	2.71
2	10.06	4.40	2.61
3	10.07	4.42	2.48
4	10.03	4.40	2.41
5	10.02	4.37	2.61
6	10.00	4.38	2.72
**Average**	**-**	**-**	**2.59 ± 0.12**

### Determination of poles and zeroes

Poles and zeroes were analytically determined from the reflection coefficient, $\:R=({Z}_{HR}-{Z}_{0})/({Z}_{HR}+{Z}_{0})$ by setting the numerator and denominator, respectively, to zero. Expanding the numerator and denominator in Fourier notation, and adopting the harmonic time dependence $\:{e}^{-i\omega\:t}$^46^, leads to a quartic equation in $\:s$ of the general form ${a}_{4}{s}^{4}+{a}_{3}{s}^{3}+{a}_{2}{s}^{2}+{a}_{1}s+{a}_{0}=0$, where the coefficients $\:{a}_{i}^{(p/z)}$ (where *p* and z indicate *‘pole’* and *‘zero’*, respectively) depend on the system parameters that dictate each element’s impedance: $\:{a}_{0}^{(p/z)}=1$, $\:{a}_{1}^{(p/z)}={R}_{M}{C}_{M}\pm\:{Z}_{0}{C}_{M}\pm\:{Z}_{0}{C}_{V}$, $\:{a}_{2}^{(p/z)}={L}_{M}{C}_{M}+{L}_{N}{C}_{M}+{L}_{N}{C}_{V}\pm\:{Z}_{0}{R}_{M}{C}_{V}{C}_{M}$, $\:{a}_{3}^{(p/z)}={L}_{N}{R}_{M}{C}_{M}{C}_{V}\pm\:{Z}_{0}{L}_{M}{C}_{M}{C}_{V}$, $\:{a}_{4}^{(p/z)}={L}_{N}{L}_{M}{C}_{M}{C}_{V}$. The quartic nature of this equation necessitates the use of algebraic methods, such as Ferrari’s solution, although modern computational tools (e.g., Mathematica) provide a more practical means of evaluation. The resulting expressions yield four roots for both poles and zeroes (Eq. 5). As expected, two of these correspond to negative real frequencies, which represent non-physical solutions, analogously to the solutions to Eqs. (3) and (4).

5 $$\:{s}^{(p/z)}=-\frac{{a}_{3}^{(p/z)}}{4{a}_{4}^{(p/z)}}\pm\:\frac{1}{2}\sqrt{{U}^{(p/z)}\mp\:\frac{{F}^{(p/z)}}{4{S}^{(p/z)}}}\mp\:\frac{{S}^{(p/z)}}{2}$$

With coefficients as below.

6 $$\:{\varDelta\:}_{0}^{(p/z)}=12{a}_{0}^{(p/z)}{a}_{4}^{(p/z)}-3{a}_{1}^{(p/z)}{a}_{3}^{(p/z)}+{{a}_{2}^{(p/z)}}^{2}$$

7 $$\begin{aligned} {\varDelta}_{1}^{(p/z)} = & -72{a}_{0}^{(p/z)}{a}_{2}^{(p/z)}{a}_{4}^{(p/z)}+27{a}_{0}^{(p/z)}{{a}_{3}^{(p/z)}}^{2}+27{{a}_{1}^{(p/z)}}^{2}{a}_{4}^{(p/z)} \\ & -9{a}_{1}^{(p/z)}{a}_{2}^{(p/z)}{a}_{3}^{(p/z)}+2{{a}_{2}^{(p/z)}}^{3} \end{aligned}$$

8 $$\:{C}^{(p/z)}=\sqrt[3]{\frac{\sqrt{{{\varDelta\:}_{1}^{(p/z)}}^{2}-4{{\varDelta\:}_{0}^{(p/z)}}^{3}}+{\varDelta\:}_{1}^{(p/z)}}{2}}$$

9 $$\:{S}^{(p/z)}=\sqrt{-\frac{2{a}_{2}^{(p/z)}}{3{a}_{4}^{(p/z)}}+\frac{{{a}_{3}^{(p/z)}}^{2}}{4{{a}_{4}^{(p/z)}}^{2}}+\frac{{C}^{(p/z)}}{3{a}_{4}^{(p/z)}}+\frac{{\varDelta\:}_{0}^{(p/z)}}{3{a}_{4}^{(p/z)}{C}^{(p/z)}}}$$

10 $$\:{F}^{(p/z)}=\frac{4{a}_{2}^{(p/z)}{a}_{3}^{(p/z)}}{{{a}_{4}^{(p/z)}}^{2}}-\frac{8{a}_{1}^{(p/z)}}{{a}_{4}^{(p/z)}}-\frac{{{a}_{3}^{(p/z)}}^{3}}{{{a}_{4}^{(p/z)}}^{3}}$$

11 $$\:{U}^{(p/z)}=\frac{{{a}_{3}^{(p/z)}}^{2}}{2{{a}_{4}^{(p/z)}}^{2}}-\frac{4{a}_{2}^{(p/z)}}{3{a}_{4}^{(p/z)}}-\frac{{C}^{(p/z)}}{3{a}_{4}^{(p/z)}}-\frac{{\varDelta\:}_{0}^{(p/z)}}{3{{a}_{4}^{(p/z)}}^{3}{C}^{(p/z)}}$$

For the CN-HR, the coefficients are analogously obtained, and given by: $\:{a}_{0}^{(p/z),CN}=1$, $\:{a}_{1}^{(p/z),CN}={\pm\:Z}_{0}{C}_{E}\pm\:{Z}_{0}{C}_{V}$, $\:{a}_{2}^{(p/z),CN}={L}_{N}{C}_{E}+{L}_{N}{C}_{V}+{L}_{E}{C}_{E}$, $\:{a}_{3}^{(p/z),CN}=\pm\:{Z}_{0}{L}_{E}{C}_{V}{C}_{E}$, $\:{a}_{4}^{(p/z),CN}={L}_{N}{L}_{E}{C}_{V}{C}_{E}$, where $\:{C}_{E}$ is the compliance of the elastomeric wall. The compliance of the elastomeric wall is given by $\:{C}_{E}=1/\left({\omega\:}_{E}^{2}{L}_{E}\right)$, with $\:{L}_{E}=4{\rho\:}_{E}{H}_{N}/\left(3\right({A}_{C}-{A}_{N}\left)\right)$ (where *A*_*C*_ is the cross-section area of the cavity and $\:{\rho\:}_{E}$ is the density of the elastomer), and $\:{\omega\:}_{E}$ is the resonance frequency of the compliant neck^48,64^.

For the P-CN-HR, the zeroes and poles are determined semi-analytically. By following the procedure described before (and introducing $\:{L}_{P}$, $\:{C}_{P}$, and $\:{R}_{P}$ as the inertance, compliance, and resistance of the elastomeric plate), the coefficients of the polynomial (which will now be of grade 6) are given by:

$$\:{{a}_{0}}^{(p/z)}=1$$

$$\:{{a}_{1}}^{(p/z)}=\frac{{R}_{P}}{{L}_{P}}\pm\:\frac{{Z}_{0}}{{L}_{N}}+\frac{{R}_{E}}{{L}_{E}}$$

$$\:{{a}_{2}}^{(p/z)}=\pm\:\frac{{R}_{P}{Z}_{0}}{{L}_{N}{L}_{P}}+\frac{{R}_{E}{R}_{P}}{{L}_{E}{L}_{P}}+\frac{{R}_{P}{Z}_{0}}{{L}_{N}{L}_{P}}\pm\:\frac{{R}_{E}{Z}_{0}}{{L}_{E}{L}_{N}}+\frac{1}{{C}_{V}{L}_{P}}+\frac{1}{{C}_{V}{L}_{N}}+\frac{1}{{C}_{V}{L}_{E}}+\frac{1}{{C}_{P}{L}_{P}}+\frac{1}{{C}_{E}{L}_{E}}$$

$$\:{{a}_{3}}^{(p/z)}=\frac{{N}_{3}}{{C}_{E}{C}_{P}{C}_{V}{L}_{E}{L}_{N}{L}_{P}}$$

$$\begin{aligned} {{N}_{3}}^{(p/z)} = & \pm\:{C}_{E}{C}_{P}{C}_{V}{R}_{E}{R}_{P}+{C}_{E}{C}_{P}{L}_{E}{R}_{P}+{C}_{E}{C}_{P}{L}_{N}{R}_{E}+{C}_{E}{C}_{P}{L}_{N}{R}_{P}+{C}_{E}{C}_{P}{L}_{P}{R}_{E}+{C}_{E}{C}_{V}{R}_{E}{L}_{N}+{C}_{P}{C}_{V}{L}_{N}{R}_{P} \\ & \pm\:{Z}_{0}\left({C}_{E}{C}_{P}{L}_{E}+{C}_{E}{C}_{P}{L}_{P}+{C}_{E}{C}_{V}{L}_{E}+{C}_{V}{C}_{P}{L}_{P}\right) \end{aligned}$$

$$\:{{a}_{4}}^{(p/z)}=\frac{{N}_{4}}{{C}_{E}{C}_{P}{C}_{V}{L}_{E}{L}_{N}{L}_{P}}$$

$$\begin{aligned} {{N}_{4}}^{(p/z)} = & {C}_{E}{C}_{P}{R}_{E}{R}_{P}+{C}_{E}{L}_{E}+{C}_{E}{L}_{N}+{C}_{P}{L}_{N}+{C}_{P}{L}_{P}+{C}_{V}{L}_{N} \\& \pm\:{Z}_{0}\left({C}_{E}{C}_{P}{R}_{E}+{C}_{E}{C}_{P}{R}_{P}+{C}_{E}{C}_{V}{R}_{E}+{C}_{V}{C}_{P}{R}_{P}\right) \end{aligned}$$

$$\:{{a}_{5}}^{(p/z)}=\frac{{C}_{E}{R}_{E}\pm\:{C}_{E}{Z}_{0}+{C}_{P}{R}_{P}\pm\:{C}_{P}{Z}_{0}+{C}_{V}{Z}_{0}}{{C}_{E}{C}_{P}{C}_{V}{L}_{E}{L}_{N}{L}_{P}}$$

$$\:{{a}_{6}}^{(p/z)}=\frac{1}{{C}_{E}{C}_{P}{C}_{V}{L}_{E}{L}_{N}{L}_{P}}$$

## Data Availability

The datasets generated and/or analysed during the current study are not publicly available due to being part of ongoing research but are available from the corresponding author on reasonable request.
